# Inhibition changes across the lifespan: experimental evidence from the Stroop task

**DOI:** 10.1186/s40359-024-01844-0

**Published:** 2024-06-07

**Authors:** Giuseppe Forte, Giovanna Troisi, Francesca Favieri, Maria Casagrande

**Affiliations:** 1 Department of Dynamic, Clinical Psychology and Health Studies, “Sapienza” University of Rome, Rome, Italy; 2 Department of Psychology, “Sapienza“ University of Rome, Rome, Italy

**Keywords:** Inhibition, Life span, Aging, Children, Adolescents, Adults, Stroop task, Executive functions

## Abstract

Individuals constantly exert inhibitory control over their thoughts and behaviors to plan actions that compete with habits and impulses. Cognitive inhibition enhances the selection of task-relevant stimuli and is closely related to neural changes that occur across the lifespan. Since few studies have focused on the entire lifespan, this study aimed to assess cognitive inhibition abilities in a sample of 425 healthy participants (age range: 7–88 years) using the Stroop task. The participants were grouped according to age into children, adolescents, young adults, adults, middle-aged adults, and older adults. A series of ANOVAs considered Group as the independent variable and Performance indices as the dependent variables. The children did not show an interference effect (Stroop effect), likely due to the lack of an automated reading process as a consequence of ongoing brain maturation. Adolescents and young adults performed significantly faster than older adults did. The results indicate that response speed reaches its peak during adolescence and young adulthood and then slightly decreases until older age. Nevertheless, when compared with the other groups, only older adults showed significant differences in the Stroop effect, suggesting that inhibitory abilities remain relatively consistent throughout adulthood but rapidly worsen in recent years due to the physiological decline in cognitive and brain functioning associated with aging.

## Introduction

The term “inhibition” refers to the ability to suppress actions or thoughts to achieve our goals [1, 2]. Inhibitory processes enable us to silence irrelevant environmental stimuli and focus on relevant information. The ability to inhibit spontaneous but contextually inappropriate actions allows goal-directed behavior and prevents automaticity from taking over [3]. Inhibition is one of the core processes of executive functions (EFs), which are high-order cognitive abilities needed for complex cognitive processing (see [4–6]. Although EFs show quite different developmental and aging trajectories [7], they all appear to reach their peak in late adolescence and early adulthood, followed by a decline in older age, reflecting changes in the prefrontal cortex throughout life. A recent study by Rodriguez-Nieto et al. [8] aimed to detect the engagement between inhibition, shifting, and working memory via a network approach, confirmed the significant differentiation in EFs from childhood to adulthood. However, there persists a dearth of attention to the inhibition evolution. Moreover, as suggested by the recent study by Ferguson and colleagues [7], the majority of studies have analyzed the differences between young and old adults, with few including a more diverse age range that encompasses children, adolescents, and middle-aged adults in the same sample to explore the age-related changes and the characteristics of the development trajectories in cognitive development further [9].

Examining the development of inhibition and its specific trajectories is relevant. The current literature suggests that inhibition, like other EFs, may be subject to physiological decline due to aging and possible pathological decline caused by multiple factors [10]. Most of these studies have compared the performance of two groups (young vs. old) on tasks typically used to assess inhibitory abilities, such as the Stroop task. However, the results of these studies were inconsistent. Some studies have indicated age-related deficits in inhibition (e.g., [11, 12]) while others have not (e.g., [13, 14]). For example, a study conducted by Gajewski et al. [15] highlighted a gradual decrease in performance on the Stroop Task with increasing age; the authors underscored slower information processing and accuracy in older adults, along with an increased intra-individual variability of speed. More surprisingly, some studies have shown that older adults perform better than young adults in inhibitory tasks [16, 17]. Attempting to overcome these inconsistencies and to further investigate the characteristics of inhibitory abilities, several authors have focused on the process of executive control maturation during aging rather than on its decline. Several researchers have compared inhibitory abilities between children and adolescents (e.g., [18, 19]). The results highlight that the basic ability to suppress a dominant behavioral response emerges in the first year of life [20] and continues to develop throughout the preschool years [21]. However, the majority of the evidence suggests that the cognitive component of inhibition, which involves the ability to suppress strong mental representations [4, 22], develops consistently throughout childhood and adolescence, reaching maturity in the first adulthood [23–25]. The developmental profile of inhibitory control is closely associated with structural and functional brain changes, particularly involving the prefrontal cortex (e.g., [26]); in fact, the prefrontal cortex is among the last to mature and among the first to deteriorate [7, 27]. However, these findings provide only a partial understanding of the development of inhibitory abilities. A substantial gap needs to be filled in the experimental paradigms that focus only on specific age groups. To investigate cognitive changes within a comprehensive framework, a recent study [7] examined the trajectories of different EFs in a large, community-based sample ranging in age from 10 to 86 years old; the results showed that inhibitory control abilities increase from adolescence to young adulthood and begin to decline slightly from approximately age 35 years. Conversely, Belghali et al. [28] found that EFs – as assessed through the Stroop Switching Card Test - appear to decline from 50 years of age. Their findings indicated an age-related decline in conflict adaptation, as evidenced by the number of errors made by midlife and older adults being higher than those of younger adults, regardless of processing speed [28]. From this point of view, the trajectory of inhibitory control can be represented by an inverted U-shaped curve, reaching its peak during adolescence and early adulthood. Although there is evidence that inhibitory control undergoes substantial changes with age, studies examining the developmental course of this executive function across the lifespan remain scarce. Therefore, the present study aimed to better understand how cognitive inhibition, as measured by the Stroop task, changes across different ages. As previously mentioned, earlier studies have focused only on understanding the association between age and inhibitory functioning [7] without analyzing and comparing differences in inhibition within each age group. This limitation prevents the clarification of inhibitory features in different developmental and aging phases. Accordingly, we want to complement these two analyses by providing a more complete answer. Specifically, we aimed to analyze the association between age and cognitive inhibition. Based on previous evidence, we expected to observe a nonlinear trend. Moreover, to gain a more nuanced understanding of the specific nature of this trend, we compared different age groups. We predicted that nonlinear trends would show a pattern characterized by the development of inhibition throughout adolescence up to early adulthood, as indicated by better performance. Furthermore, we hypothesized that an age-related decline would start in middle adulthood and continue until older adulthood.

## Method

### Participants

Four hundred and twenty-five healthy Italian individuals participated in the studies. The recruitment strategies used varied depending on the age of the participants. Specifically, announcements were distributed through the main social media platform and published on the Laboratory website to recruit participants from the general population older than 18 years. Children and adolescents were recruited through interactions with some local educational institutions; the announcement was then forwarded to the parents of the children and adolescents who could authorize their participation. The voluntary nature of the participation was clearly stated in the description of the study written on the announcements, as was the duration of the experimental procedure (15 min), the inclusion criteria (i.e., age between 7 and 90 years, free of psychiatric, cognitive or clinical conditions) and the exclusion criteria (i.e., not meeting the inclusion criteria).

A semi-structured face-to-face interview was conducted to assess the presence of the inclusion and exclusion criteria. Participants aged 60 years and older were administered the Mini Mental State Examination (MMSE; [29]), and only those with a MMSE score of 28 or higher were included. This criterion allowed us to exclude individuals with mild or severe cognitive decline.

The final sample of participants ranged in age from 7 to 88 years. In line with our aims, participants were divided into six groups: (1) 55 children (mean age: 7.96, SD: 0.92, range: 7–11; 31 females); (2) 68 adolescents (mean age: 17.34, SD: 1.43, range: 16–20 years; 49 females); (3) 77 young adults (mean age: 25.31, SD: 3.46, range: 21–34; 43 females); (4) 98 adults (mean age: 49.67, SD: 4.93, range: 37–55; 72 females); (5) 82 middle-aged adults (mean age: 60.20, SD: 2.97, range: 56–65; 60 females); and (6) 46 older adults (mean age: 71.50, SD: 5.28, range: 66–88; 20 females). Similarly to other studies [7, 30], the participants were stratified by age range. As the literature uses different age ranges for young adults and middle-aged adults - with the vast majority of studies defining older individuals as those aged 65 and above (e.g., [15]) - we divided the sample into smaller age groups in order to capture potential nuances in cognitive functioning during adulthood [7, 30].

### Measurements

#### Anamnestic sociodemographic interview

Each participant completed a semistructured sociodemographic interview to collect data on age, sex, education level, marital status, history and presence of clinical/psychiatric conditions, and history of head trauma. The interview was used to verify whether the inclusion criteria were met.

#### Assessment of inhibitory control

All participants completed a computerized version of the Stroop task [22]. The task involved reading colored words (Font: Courier New; Font size: 60; colors: yellow, red, blue, green) that referred to the colors YELLOW, RED, BLUE, and GREEN. Each word was presented in two conditions: the congruent condition, where the word was written with the ink color matching its semantic meaning (e.g., BLUE written in blue ink); and the incongruent condition, where the ink color in which the word was presented did not match the semantic meaning of the word (e.g., BLUE written in red ink). Participants were instructed to press the key corresponding to the ink color (A = red; S = green; K = blue; L = yellow; each key had a label of the corresponding color) as quickly and accurately as possible. A practice block of 15 trials with feedback on correct performance was presented at the beginning of the task. Following the practice block, participants completed a block of 120 randomly presented trials (60 for each condition). An initial fixation cross (400 ms) was presented on the screen before each trial [22].

The target stimulus duration was 3000 ms or until the participant’s response. Reaction times (RTs) and accuracy were recorded. The Stroop effect was computed as follows: mean RTs incongruent trials - mean RTs congruent trials.

### General Procedure

Each participant underwent a single experimental session. At the beginning of the session, participants signed a written informed consent. Anamnestic data (e.g., age, sex, educational level) were collected through a semistructured sociodemographic face-to-face interview (for children, the interview was conducted with their parents), after which the Stroop task was administered. Before completing the behavioral task, the children were tested if they had sufficient understanding/reading ability to complete the task. The whole experimental procedure lasted approximately 15 min and took place in a quiet laboratory room at the University of Rome “Sapienza”.

### Statistical analysis

Reaction times (RTs) greater than 200 ms for correct responses and accuracy were considered dependent variables. To normalize the distribution, RTs were transformed into natural logarithms (ln) [31]. The percentage of RTs slower than 200 ms or delayed responses (over 3000 ms) was less than 1%. Consequently, no participant was excluded from the analysis. The Stroop effect was obtained by subtracting the ln RTs of congruent trials from the ln RTs of incongruent trials.

A series of regressions were performed to examine the correlation between age and inhibition. The regressions considered age as an independent variable, the RTs of congruent and incongruent trials, and the Stroop effect as dependent variables. Linear and quadratic orthogonal coefficients were used to assess the relationship between age and inhibition. To gain a more comprehensive understanding of this association, five separate correlations between age and performance on the task (both RTs and accuracy) were conducted separately for each group.

Furthermore, mixed design analyses of variance (ANOVAs) Group (Children, Adolescents, Young adults, Adults Middle-aged adults, Older adults) × Congruency (Congruent, Incongruent) separately considered RTs and accuracy as dependent variables. Given the studies indicating a sex difference in the Stroop task [32] a preliminary ANOVA was conducted to examine the effect of sex. However, the main effect and interactions were not significant (F < 1), and thus this factor was not included in the final analyses. Another ANOVA considered the Group as the independent variable and the Stroop effect as the dependent variable. Due to the exploratory nature of this study, post hoc analysis with Bonferroni correction was adopted to analyze the main effects and the interactions. For all ANOVAs, partial eta squared was employed as a measure of effect size. A value of *p* < 0.05 was considered to indicate statistical significance. The analyses were performed using Jasp statistical software.

## Results

### Sociodemographic characteristics

The sociodemographic characteristics of the sample (i.e., sex, age, education level) are summarized in Table 1.

**Table 1:** Characteristics of the samples

Age Group	*N* (F/M)	Age range	Mean Age (±SD)	Years of education
*Children*	55 (31/24)	7–11	7.96 (0.92)	2.80 (0.70)
*Adolescents*	68 (49/19)	16–20	17.34 (1.43)	12.10 (1.12)
*Young Adults*	77 (43/34)	21–34	25.31 (3.46)	17.10 (2.32)
*Adults*	98 (72/26)	37–55	49.67 (4.93)	15.40 (3.96)
*Middle-aged adults*	82 (60/22)	56–65	60.20 (2.97)	17.10 (4.39)
*Older adults*	46 (20/26)	66–88	71.50 (5.28)	16.10 (2.32)

SD: Standard deviation

### Stroop Task

#### Linear Regression models

Three separate regression analyses were conducted to investigate and confirm the predictive role of age on performance on the task (i.e., congruent trials, incongruent trials, Stroop effect). The predictive model included both age and age-squared values, according to Ferguson et al. [7].

**Fig. 1:**
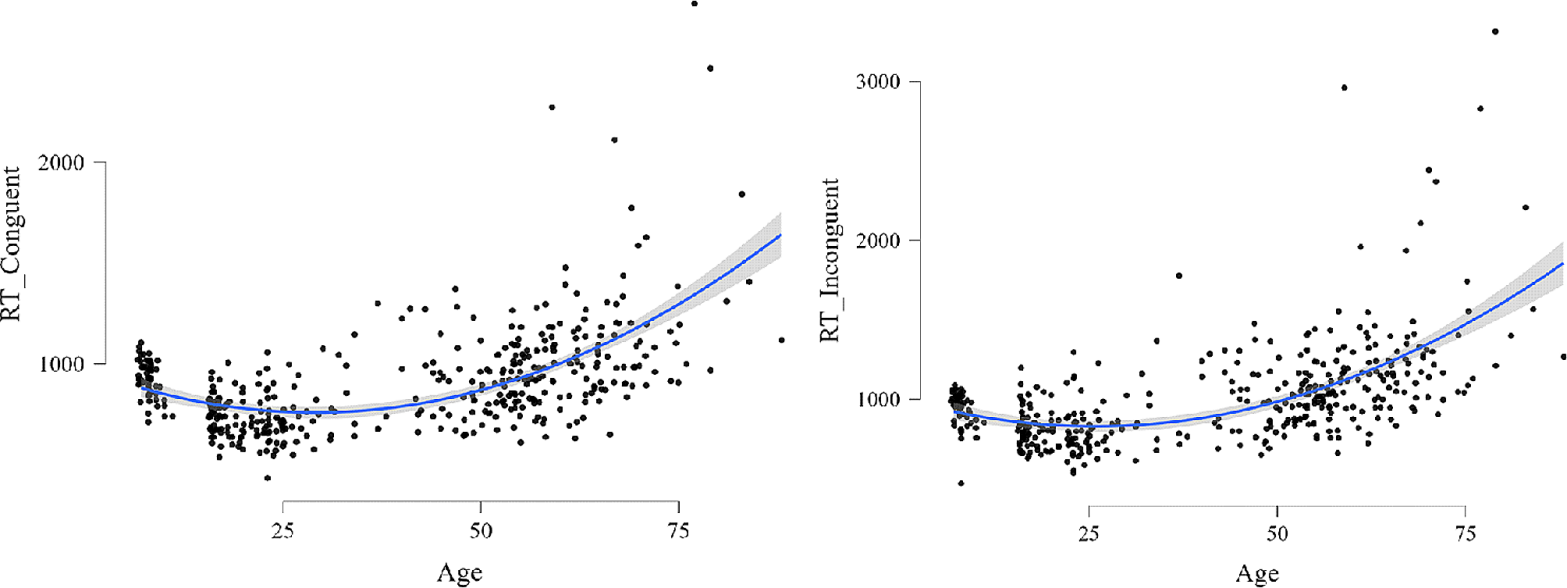
Relationship between age and executive Stroop Performance (Reaction time; RT).

Specifically, a significant predictive role of both linear and quadratic coefficients of age on the ln-transformed RTs of the Stroop Task emerged for both Congruent (R^2^ = 0.36, F(2,422) = 117, *p* < 0.001; linear: b_s_=-1.26, *p* < 0.001; quadratic: b_s_=1.77, *p* < 0.001) and Incongruent Trials (R^2^ = 0.39, F(2,422) = 132, *p* < 0.001; linear: b_s_=-0.94, *p* < 0.001; quadratic: b_s_=1.51, *p* < 0.001), as well as for the Stroop Effect (R^2^ = 0.084, F(2,422) = 19.3, *p* < 0.001; linear: b_s_=0.71, *p* = 0.001; quadratic: b_s_=-0.45, *p* = 0.04; see Fig. 1).

### Pearson correlation

The separate Pearson correlation results revealed different patterns according to age group (Table 2). Specifically, a significant negative correlation between RTs and age was found in children (congruent: *r*= -0.55; *p* < 0.001; incongruent *r*= -0.40; *p* < 0.002), while a positive correlation was found in young adults (congruent *r* = 0.26; *p* < 0.02) and older adults (congruent *r* = 0.30; *p* = 0.04; incongruent *r* = 0.33; *p* = 0.02). No other significant correlations were reported for RT. Furthermore, no significant correlation was highlighted in terms of accuracy.

**Table 2:** Pearson correlation coefficients of the association between age (in each subgroup) and Stroop task performance

	Overall sample	Children	Adolescents	Young Adults	Adults	Middle-aged Adults	Older Adults
RTs Congruent trials	0.45***	-0.55***	-0.22	0.26*	0.02	0.06	0.30*
RTs Incongruent trials	0.50***	-0.40***	-0.12	0.21	0.02	0.11	0.33*
RTs Stroop Effect	0.36***	0.09	0.13	-0.02	0.01	0.17	0.21
%ACC Congruent trials	-0.05	0.11	0.09	-0.09	-0.12	0.02	-0.10
%ACC Incongruent trials	-0.05	0.06	0.01	-0.03	-0.12	-0.01	0.03
%ACC Stroop Effect	-0.01	-0.01	-0.13	0.14	-0.04	-0.12	0.08

* *p* < 0.05, ** *p* < 0.01, *** *p* < 0.001

### Between-group differences in the congruent and incongruent conditions

#### Reaction times

The main effects of Group (F(5,420) = 43.78, *p* < 0.001; η^2^ = 0.32) and Congruence (F(1,420) = 329.51, *p* < 0.001, η^2^ = 0.03) were significant. Faster RTs were reported in the congruent condition.

Considering between-group differences in RTs, adolescents were faster than all the groups (children (t = 3.55; *p* = 0.006), adults (t = 4.93; *p* < 0.001), middle-aged adults (t = 7.50; *p* < 0.001), and older adults (t = 12.18; *p* < 0.001) but did not differ from young adults (t < 1; *p* = 1.00). The children were slower than younger adults (t = 3.81; *p* = 0.002), middle-aged adults (t = 3.36; *p* = 0.01), and older adults (t = 8.41; *p* < 0.01) but not adults (t < 1; *p* = 1.0). Younger adults were faster than adults were (t = 5.30; p 0,0001), middle-aged adults were (t = 7.93; p 0,0001), and older adults were (t = 12.64 p 0,0001). Finally, adults were faster than middle-aged (t = 3.02; *p* = 0.04) and older adults (t = 8.66; *p* < 0.0001).

The Group × Congruency interaction was significant (F(5,420) = 14.87; *p* < 0.001; η^2^ = 0.006). Post hoc analyses revealed that, while no differences emerged between the congruent and incongruent conditions (t(420) = 0.64; *p* = 0.52), slower RTs in the incongruent condition than in the congruent condition were reported in all the groups: (i) adolescents (t(1,420) = 6.24; *p* < 0.001); (ii) young adults (t(1,420) = 6.76; *p* < 0.001); (iii) adults (t(1,420) = 9.48; *p* < 0.001); (iv) middle-aged (t(1,420) = 10.90; *p* < 0.001); and (v) older adults (t(1,420) = 11.33; *p* < 0.001).

Moreover, interesting results emerged for both congruency conditions. Specifically, in the congruent condition, the children had slower RTs than did the adolescents (t = 4.32, *p* = 0.001) and young adults (t = 4.61, *p* < 0.001), while they reported faster RTs than did the older adults (t= -6.35, *p* < 0.001). No significant differences emerged in performance between Children and Adults (t < 1, *p* < 1.0) or between Children and Middle-Aged adults (t=-1.78, *p* = 0.60). Adolescents and young adults did not significantly differ in RTs in the congruent condition (t < 1; *p* = 0.99). However, both groups of participants had faster RTs than did Adults (Adolescents vs. Adults: t= -4.50, *p* < 0.001; Young Adults vs. Adults: t= -4.87, *p* < 0.001); Middle Aged (Adolescents vs. Middle Aged: t= -6.66, *p* < 0.001; Young Adults vs. Middle Aged: t= -7.08, *p* < 0.001); and Older adults (Adolescents vs. Older adults: t= -10.75, *p* < 0.001; Young Adults vs. Older adults: t= -11.17, *p* < 0.001). Older adults also reported slower RTs than did both adults (t = 7.50; *p* < 0.001) and middle-aged adults (t = 5.19, *p* < 0.001). The means and standard deviations of the RTs are shown in Table 2 (see Fig. 1).

In the incongruent condition, post hoc analyses showed that children had faster RTs than did both middle-aged (t=-4.76, *p* < 0.001) and older adults (t=-10.02, *p* < 0.001). However, they did not differ from either adolescents (t = 2.60, *p* = 0.63) or young adults (t = 2.81, *p* = 0.33); moreover, no significant differences emerged between adolescents and young adults (t < 1 *p* = 1.0). Both adolescents and young adults reported faster RTs in the incongruent condition than did adults (adolescent vs. adults: t= -5.09, *p* < 0.001; young adults vs. adults: t= -5.44, *p* < 0.001), middle-aged adults (adolescent vs. middle-aged adults: t= -7.93, *p* < 0.001; young adults vs. middle-aged adults: t= -8.36; *p* < 0.001) and older adults (adolescents vs. older adults: t= -12.96, *p* < 0.001; young adults vs. older adults: t= -13.41, *p* < 0.001). Older adults were the slowest group in incongruent trials, compared to both adults (t= -9.35, *p* < 0.001) and middle-aged adults (t= -6.37, *p* < 0.001). The means and standard deviations of the RTs are shown in Table 3.

**Table 3:** Means and standard deviations of RTs and accuracy in congruent and incongruent trials and in the Stroop test

	Children	Adolescents	Young adults	Adults	Middle-aged adults	Older adults	F	*p*	⎜^2^_*p*_
	*M*	*M*	*M*	*M*	*M*	*M*			
Reaction Time									
*Congruent*	952.42 (94.12)^bcf^	744.15 (101.11)^def^	736.90 (137.88)^def^	908.71 (175.02)^f^	997.36 (226.48)^f^	1219.05 (417.94)	43.90	< 001	0.34
*Incongruent*	934.66 (106.66)^ef^	825.43 (126.32)^def^	819.53 (169.25)^def^	1011.50 (210.45)	1126.56 (297.42)	1398.36 (518.76)	41.16	< 0.001	0.33
*Stroop Effect*	9.24 (93.92)	81.23 (52.90)^af^	82.63 (72.20)^af^	102.79 (90.89)^af^	129.198 (122.41)^a^	179.32 (208.28)^a^	14.87	< 0.001	0.15
Accuracy									
*Congruent*	94.33 (8.62)	97.58 (6.28)	97.34 (9.89)	91.94 (18.01)	95.25 (14.82)	97.19 (10.27)	2.46	0.03	0.03
*Incongruent*	89.61 (14.41)	95.45 (7.24)	95.85 (10.68)	90.97 (17.83)	93.73 (16.87)	90.26 (21.89)	2.08	0.07	0.02
*Stroop Effect*	-4.73 (12.57)	-2.14 (3.76)^f^	-1.49 (4.36)^f^	-0.98 (2.99) ^f^	-1.52 (4.44) ^f^	-6.93 (19.81)	4.24	< 0.001	0.05

^a^Significant differences compared to children; ^b^ Significant differences compared to adolescents; ^c^ Significant differences compared to young adults; ^d^Significant differences compared to adults; ^e^ Significant differences compared to middle-aged adults; ^f^ Significant differences compared to older adults

#### Accuracy

The main effects of Group (F(5,420) = 2.03, *p* = 0.7; η^2^ = 0.02) were not significantly different, while the main effect of Congruency (F(1,420) = 47.56, *p* < 0.001; η^2^ = 0.01) was significantly greater, indicating greater accuracy in congruent trials than in incongruent trials.

The Group x Congruency interaction was significant (F(5,420) = 4.24; *p* < 0.001; η^2^ = 0.005), and post hoc analyses revealed a significant difference between congruent and incongruent trials in children (t = 4.07; *p* = 0.004) and older adults (t = 5.46; *p* < 0.0001), with greater accuracy in the congruent condition than in the incongruent condition. No other significant differences were highlighted (all t < 3.10; all *p* > 0.13).

#### Stroop effect (RT)

One-way ANOVA was used to analyze the differences between groups in terms of the Stroop effect on RTs (F(5,420) = 14.87, *p* < 0.001, η²_p_= 0.15). Post hoc tests highlighted that children reported a lower Stroop effect than did all the other groups (adolescents: t= -3.70, *p* = 0.003; young adults: t= -3.89, *p* = 0.002; adults: t= -5.17, *p* < 0.001; middle-aged adults: t= -6.41, *p* < 0.001; older adults: t= -7.93, *p* < 0.001). In contrast, older adults had a greater Stroop effect than did all the other groups (adolescents: t = 4.78, *p* < 0.001; young adults: t = 4.84, *p* < 0.001; adults: t = 3.99, *p* = 0.001), except for the middle-aged group (t = 2.53, *p* = 0.11; see Table 2).

#### Stroop effect (accuracy)

The Stroop effect calculated for %Acc showed a significant difference between the groups (F(5,420) = 4.24, *p* < 0.001; η²_p_=0.05). Specifically, post hoc analysis revealed a greater Stroop effect in older adults than in adolescents (t = 2.92, *p* = 0.04), young adults (t = 3.39, *p* = 0.01), adults (t = 3.87, *p* = 0.002), and middle-aged adults (t = 3.41, *p* = 0.009), but not with respect to children (t = 1.28, *p* = 0.79; see Table 2).

## Discussion

The present study aimed to determine how cognitive inhibition develops and evolves with age and to further examine the U-shaped trend suggested by previous studies [7]. Since few studies have focused on the entire lifespan to delineate the inhibition trajectories of inhibition, we investigated performance on the Stroop task from late childhood to old age in a large sample of healthy individuals. According to the literature on the Stroop task, this tool allows the assessment of different features of cognitive components involved in selective attention and inhibitory control. Specifically, adequate performance on the Stroop task is characterized by slower reaction times in incongruent trials than in congruent trials. This is interpreted as the expression of selective inhibition, dampening the fast automatic activation associated with word reading [33]. Moreover, Stroop interference, which refers to the difference in reaction times or accuracy between incongruent and congruent conditions (known as the Stroop effect), is typically considered an indicator of inhibitory ability.

Considering the characteristics intrinsic to the task, our results showed (a) faster reaction times in congruent than in incongruent trials in the whole sample, except for the children’s group; (b) a U-shaped distribution of reaction times in both congruent and incongruent trials, taking into account the age groups; and (c) different patterns of Stroop interference in children, middle and older adults. Accordingly, we assumed that cognitive inhibition adaptively increases from childhood to young adulthood, followed by a slight decline starting at approximately the age of 35, confirming the hypothesized inverted U-shaped curve. These findings are consistent with previous evidence [7, 27] and provide interesting insight into the developmental trajectories of inhibitory control. In fact, although studies have largely addressed the substantial changes in inhibitory control that occur in childhood [34–36] and in late adulthood [37, 38] little evidence has considered changes across the lifespan [39]. Typically, studies comparing only two (or three) samples, such as young versus older adults (e.g [40]) or children versus adolescents versus young adults (e.g [41]), could be useful for highlighting age differences but not for defining the developmental trajectories of inhibitory abilities. However, since interesting results emerge from group comparisons, to provide a comprehensive overview, we discuss below the possible explanations for the age-related changes from childhood to older age.

From a developmental perspective, the slower reaction times observed in children compared to adolescents and young adults could be ascribed to brain maturation, particularly in prefrontal areas involved in attentional and cognitive control [4]. Interestingly, our results did not show a difference between congruent and incongruent conditions in children, suggesting that Stroop interference was substantially absent. It is noteworthy that while the difference in the general slowness of children compared to that of the other groups is consistent with previous findings, our children did not exhibit an interference effect. This result contrasts with previous studies that, in some cases, reported greater interference in children than in adults due to their still in progress in automating reading ability. These contrasting results should be considered in light of the specific feature of inhibition assessed by the Stroop Task, namely the integration of active attention and top-down control [42]. Further studies should consider the possibility of comparing different tasks for inhibition (e.g., Stroop Task, Go-No/Go Task, Stop Signal Task) [23]. Other evidence has shown similar results in older samples, confirming slower reaction times and lower accuracy in incongruent trials than in neutral or congruent trials (for a meta-analysis, see [43]). The discrepancy between our results and those of other studies may be ascribed to the peculiarities of the group. During childhood, the brain undergoes rapid changes that significantly impact cognitive development and related performance. These changes can be observed even in the case of little difference in growth, such as a few months. The correlational analyses confirmed the subgroup heterogeneity of performance that may have affected the results. In fact, significant negative correlations between age and reaction times were observed in both the congruent and incongruent trials in this group. Although these results may be inconsistent with the slower performance of children than of individuals in other age groups (except older adults), they may confirm a degree of immaturity in the inhibitory response. This immaturity is expressed in both congruent and incongruent trials, reducing the interference effect typically reported by the task. Moreover, the accuracy analysis revealed significant differences between congruent and incongruent trials. These findings could indicate that children point out a trade-off effect, resulting in faster reaction times at the expense of lower accuracy. Specifically, accuracy decreased substantially in trials that required more inhibition than in those that did not. These results confirm the findings of inhibitory control of incongruent stimuli in a flanker task in 3- to 6-year-old children [36].

The similar performance of adolescents and younger adults suggests that the development of the inhibition process and brain specialization begins after the age of twelve and persists until young adulthood. This finding is consistent with brain imaging studies showing that changes in performance during adolescence are associated with brain development. Consequent cognitive maturation is linked to progressive increases in the activation of prefrontal brain regions and their connections that mediate top-down control in the context of inhibition [44–48]. Furthermore, the results obtained in these groups can be explained by the increasing cross-network integration of the cingulo-opercular/salience network, which is involved in inhibitory responses and tends to increase with age [49].

Finally, it is unsurprising that older adults exhibit slower reaction times and greater Stroop interference. Additionally, there was a difference in accuracy between congruent and incongruent trials, which suggested that after 65 years, inhibition also starts to decrease accuracy (e.g [50]). However, the precise mechanisms responsible for the longer latencies and decreased accuracy in this group are unclear. Childhood is characterized by the underdevelopment of brain networks, while late adulthood has been associated with increased deterioration in different brain regions, suggesting the general slowing hypothesis [51]. Several studies have shown that older adults have an increased Stroop effect compared to young adults [52]. This difference is attributed to age-related changes in processing speed and slowing reaction times, as well as to brain changes in frontal neural circuitry and neurobiological functioning [53]. Behavioral evidence suggests that age-related increases in Stroop interference are partially attributable to both general slowing and age-related changes in task-specific processes, such as cognitive inhibition [54–56]. These interpretations are supported by correlational analysis that showed a positive correlation between performance and age, suggesting a faster deterioration in performance after age 65, expressed by the time delay. This association is specific to this age group but is not evident in earlier stages when the inhibitory decline appears more gradual. However, our results contrast those of a meta-analysis that demonstrated no inhibitory deficits at older ages when using Stroop tasks [3]. However, further studies are needed to clarify these inconsistent results and related features.

### Limitations

A few limitations need to be addressed regarding the present study. Despite the use of a large sample, the characteristics of the sample and the methods used did not allow for a full understanding of the nature of the relationship between inhibition and aging, especially in elderly people. Future studies should consider an in-depth examination of the neuropsychological condition of elderly individuals, given the possible presence of cognitive decline as a pathological risk factor associated with aging. Indeed, the adults involved in this study did not report symptoms of cognitive decline. However, other cognitive functions (e.g., global cognitive functioning, working memory abilities, etc.) were not examined. Thus, mild cognitive impairment, i.e., a mild form of cognitive decline that does not affect daily functioning, could have influenced our results.

Moreover, the choice to use the Stroop task could present some limitations, even if it allows us to compare our results with the large body of literature in the field, including changes in performance related to different life stages. However, it is important to note that no single task can exhaustively measure all components of EFs, including inhibition. Inhibition tasks are rarely focused purely on this process. Furthermore, there is a debate about the validity of the Stroop task for measuring cognitive inhibition due to the task impurity phenomenon. We used the Stroop task to assess both selective attention and cognitive inhibition; however, it is important to consider this limitation when interpreting the results. Moreover, it would be interesting to analyze other cognitive tasks to explore further features of inhibitory control, such as motor inhibition components. To achieve this, one can compare cognitive and motor inhibition using a Stroop task and a go/no-go task. Although these tasks assess inhibition, some notable differences can lead to different results [57]. To examine the development of cognitive inhibition as a function of age, one can compare performance on a Stroop task and a Flanker task. The Flanker task assesses inhibitory control of conflicting information and is a highly sensitive measure of executive inhibitory control in preschoolers (e.g [58]), school-age children (e.g [59, 60]), adolescents (e.g [61]), young adults (e.g [62]), middle-aged adults (e.g [63]), and older adults (e.g [40]). Assessing inhibitory control throughout the lifespan using various tests may help to resolve some of the inconsistencies in the literature. This approach can also provide insight into which aspects of this executive function are still developing during childhood and to better determine the trajectory of its decline from adulthood to old age.

## Conclusions

The present study aimed to understand changes in inhibition across the lifespan. The results indicate a U-shaped development of this core executive function, demonstrating that individuals may manage environmental stimuli and goal-directed behaviors differently at different stages of life. Full maturation of the brain and the resulting optimal expression of cognitive functioning are typically observed in young and middle-aged adults. This result is a relevant point that may explain why developmental studies in cognitive science emphasize the importance of monitoring the preceding (childhood and adolescence) and following (older age) phases of life. This approach would enable better functionality while maintaining adaptability for daily functioning as long as possible.

## Data Availability

available on request.
